# Postural instability in an immersive Virtual Reality adapts with repetition and includes directional and gender specific effects

**DOI:** 10.1038/s41598-019-39104-6

**Published:** 2019-02-28

**Authors:** Per-Anders Fransson, Mitesh Patel, Hanna Jensen, Michèle Lundberg, Fredrik Tjernström, Måns Magnusson, Eva Ekvall Hansson

**Affiliations:** 1 Department of Clinical Sciences, Lund University, Lund, Sweden; 2 Division of Brain Sciences, Imperial College London, London, United Kingdom; 3 Department of Health Sciences/Physiotherapy, Lund University, Lund, Sweden

## Abstract

The ability to handle sensory conflicts and use the most appropriate sensory information is vital for successful recovery of human postural control after injury. The objective was to determine if virtual reality (VR) could provide a vehicle for sensory training, and determine the temporal and spatial nature of such adaptive changes. Twenty healthy subjects participated in the study (10 females). The subjects watched a 90-second VR simulation of railroad (rollercoaster) motion in mountainous terrain during five repeated simulations, while standing on a force platform that recorded their stability. The immediate response to watching the VR movie was an increased level of postural instability. Repeatedly watching the same VR movie significantly reduced both the anteroposterior (62%, p < 0.001) and lateral (47%, p = 0.001) energy used. However, females adapted more slowly to the VR stimuli as reflected by higher use of total (p = 0.007), low frequency (p = 0.027) and high frequency (p = 0.026) energy. Healthy subjects can significantly adapt to a multidirectional, provocative, visual environment after 4–5 repeated sessions of VR. Consequently, VR technology might be an effective tool for rehabilitation involving visual desensitisation. However, some females may require more training sessions to achieve effects with VR.

## Introduction

When we move and interact with the environment, the central nervous system (CNS) must detect and counter egocentric body movements produced by sensory information from physical or visual motion. An everyday example is the illusion of self-motion that we may experience when looking at a moving train or moving scenery. Movement of a succession of vehicles or objects across our visual field produces optically driven nystagmus, and an apparent sense of oneself being in motion that gives rise to a disturbance of postural control^1,2^. The CNS is continuously prepared to counter such imbalances to avoid potential falls^3^, but how quickly the characteristics of motor control change and what kind of adjustments the CNS utilizes to better handle visual motion is largely unknown.

In order to study how we utilize adaptive reweighting processes to handle conflicting visual information, provocative visual motion can be produced without physical motion for research purposes by virtual reality (VR). VR involves real-time simulation and interactions between sensory, motor and cognitive channels. When the user moves their head, the VR device determines the new direction of gaze and recreates the visual scene from the user’s new point of view in 3D space. Hence, VR can be set up to be strongly immersive, in that the environment appears real and three-dimensional, to induce reliable ego-motion. This is particularly useful experimentally as VR can induce motor responses to an unexpected veridical virtual input. In fact, VR simulations of riding a rollercoaster can produce such a strong illusion of movement that it causes motion sickness. VR, therefore, provides an ideal environment to study the balancing strategies used for addressing abhorrent visual flow, and for studying the properties of the adaptation processes initiated by repeated exposure to the same visual scenario^4,5^. If humans are repeatedly exposed to visual motion, the CNS may, after a while, find it necessary to adjust the processing and integration of sensory information sources to reduce postural instability^4^. Interestingly, adaptive postural skills to sensory conflicts are reduced in individuals susceptible to motion sickness, specifically seasickness. Previous investigations have shown that seasick individuals experience higher levels of low frequency postural movements when standing upright^6^, especially in the lateral plane^7,8^. As visual motion is a particularly provocative and reliable stimulus for motion sickness, we wanted to test whether low and high frequency postural responses adapt during quiet stance in a provocative VR environment. We also explored gender effects, as a higher incidence of motion sickness and postural sway has been reported in females over males in VR experiments^9^.

## Results

Twenty healthy subjects participated in the study (10 females). Subjects watched a 90-second VR simulation of a railroad (rollercoaster) motion in mountainous terrain during five repeated simulations, while standing on a force platform that recorded their stability. For details, see the Methods section presented after the Discussion section.

### Stability during repeated Virtual Reality sessions

**Figure 1:**
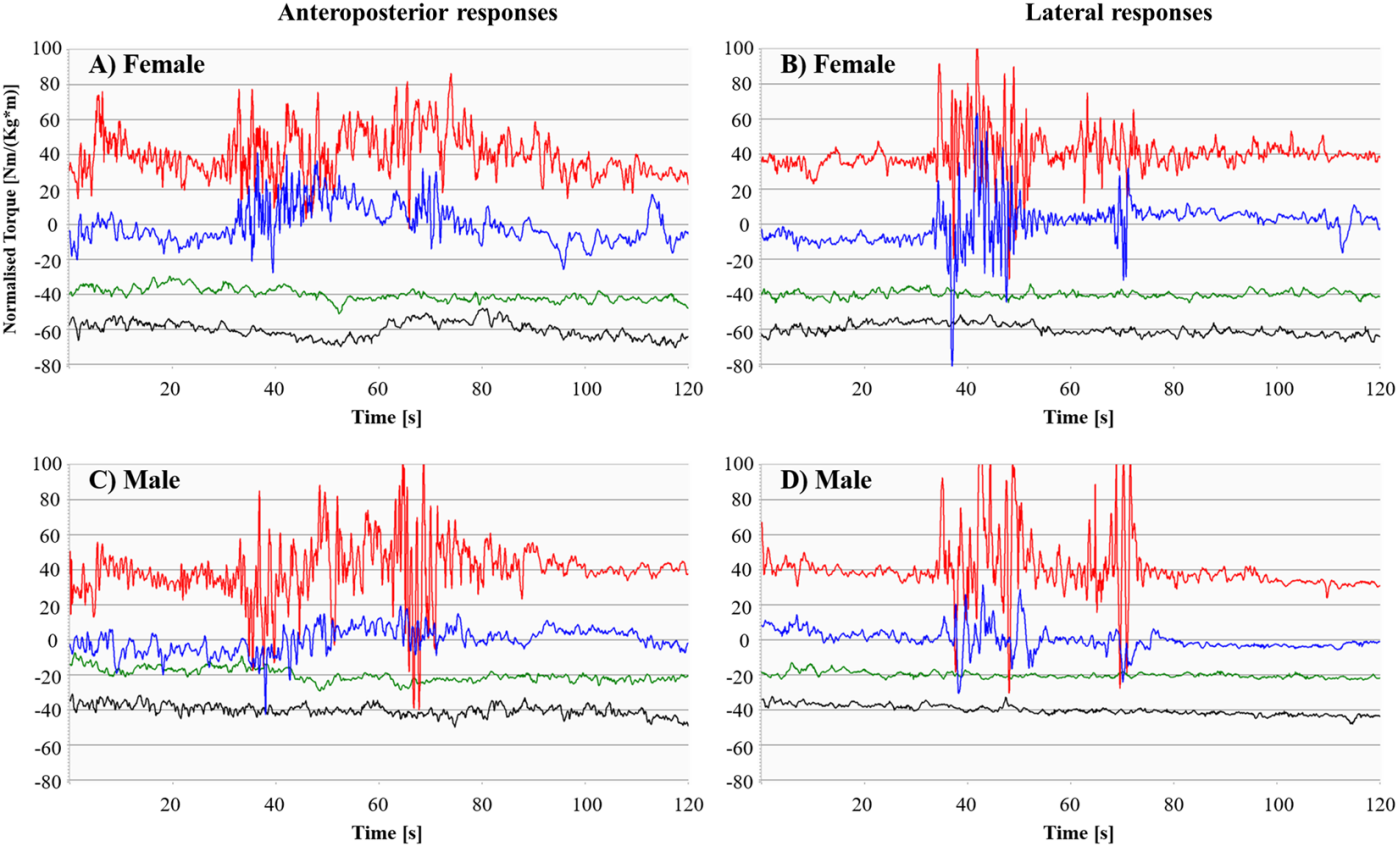
Simultaneous force platform recordings of anteroposterior and lateral responses. Stability recordings of a female (**A**,**B**) and male (**C**,**D**) test subject during the first VR session (red), fifth VR session (blue), Quiet stance with eyes open (green) and Quiet stance with eyes closed (black). The recordings are presented with the identical scales, but the y-axis has been altered to enhance visibility. The presented data is normalised for differences in height and weight.

The subjects typically responded to viewing the rollercoaster VR stimuli by making multidimensional reactive body movements (Fig. 1). Hence, travelling through a sharp curve to the right in the rollercoaster VR movie caused the subjects to both lean to the left and slightly forward, as recorded by the force platform. Both the male and female subject displayed in Fig. 1 responded strongly when watching the VR movie the first time. Both subjects also showed better postural control during the fifth VR session but only the female subject continued to respond strongly to the VR stimuli.

### Effects of Repetition and Gender on the stability during repeated VR sessions

During VR perturbations, repeatedly watching the same movie significantly reduced the energy used within all spectral ranges investigated in anteroposterior direction (p < 0.001) (Table 1). The interaction between main factors Repetition x Gender revealed that females adapted more slowly to the VR stimuli as reflected by higher use of high frequency energy (p = 0.036).

**Table 1:** General effects on the stability from repeated VR sessions.

Virtual Reality stability^a^	Repetition^b^	Gender^b^	Repetition x Gender^b^
**Anteroposterior**			
Total	**<0**.**001 [45**.**3]**	0.120 [2.7]	0.394 [0.8]
**<**0.1 Hz	**<0**.**001 [21**.**8]**	0.187 [1.9]	0.253 [1.4]
>0.1 Hz	**<0**.**001 [70**.**5]**	0.173 [2.0]	**0**.**036 [5**.**1]**
**Lateral**			
Total	**<0**.**001 [28**.**8]**	0.105 [2.9]	**0**.**002 [12**.**5]**
**<**0.1 Hz	**0**.**002 [13**.**8]**	0.141 [2.4]	**0**.**017 [6**.**9]**
>0.1 Hz	**<0**.**001 [52**.**0]**	0.106 [2.9]	**0**.**023 [6**.**1]**

^a^Repeated measures GLM ANOVA analysis of how the stability was affected by main factors “Repetition” and “Gender” alone and by their main factor interactions during the VR sessions.

^b^p-values and [F-values]. The notation “<0.001” means that the p-value is smaller than 0.001.

Similarly, repeatedly watching the same movie significantly reduced the total (p < 0.001), low frequency (p = 0.001) and high frequency (p < 0.001) energy used in lateral direction (p ≤ 0.002). However, the interaction between the main factors Repetition x Gender revealed that females adapted more slowly to the VR stimuli as reflected by higher use of total (p = 0.002), low frequency (p = 0.017) and high frequency (p = 0.023) energy in lateral direction.

**Figure 2:**
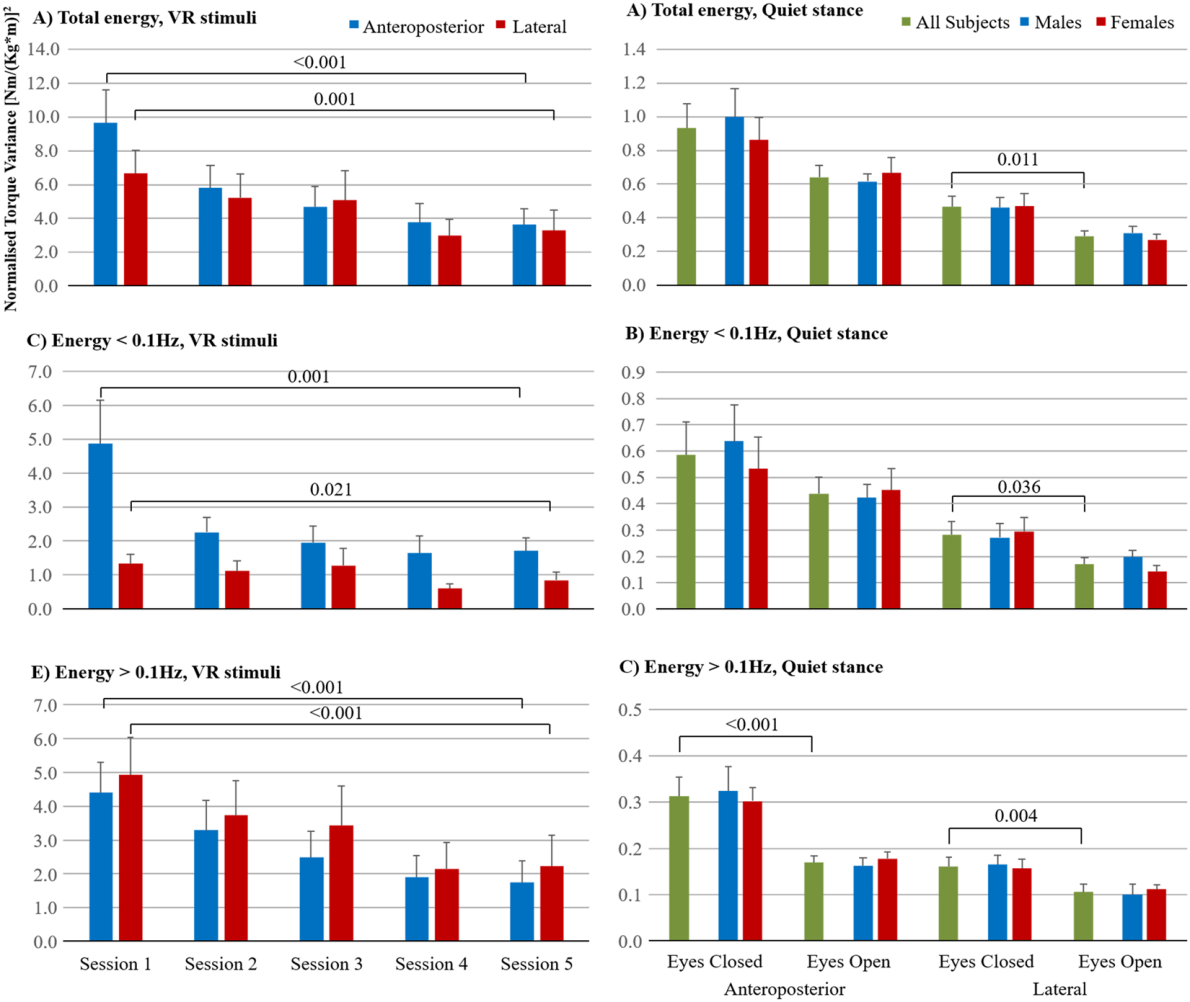
Stability during repeated VR sessions and during quiet stance. Normalised torque variance values, reflecting used energy, during repeated VR stimuli sessions and during the control tests with eyes closed and eyes open (mean and SEM values). Within all spectral ranges investigated, the subjects were able to gradually reduce the energy used, see figure (**A**,**C**,**E**). Moreover, in the control tests the subjects used less energy with eyes open and in lateral direction, see figure (**B**,**D**,**F**). The postural control responses during the VR sessions were predominantly above 0.1 Hz and of similar sizes in anteroposterior and lateral direction, which is atypical for the postural control performance found during quiet stance. No statistical differences were found between genders during the control tests. Horizontal lines represent significant difference between the first and last VR session, with the p-values for the comparisons presented above the lines.

Post-hoc analyses were performed to determine the adaptation capacity, in terms of less energy used after five repeated VR sessions, in the entire test subject group. The adaptation capacity in the entire group were significant in total (p < 0.001), low frequency (p = 0.001) and high frequency (p < 0.001) energy used in anteroposterior direction, and in total (p = 0.001), low frequency (p = 0.021) and high frequency (p < 0.001) energy used in lateral direction (Fig. 2, Table 2).

**Table 2:** Adaptive stability changes to VR and performance reached after adaptation compared to the control test quiet stance with Eyes Open.

Stability changes	p-value^a^	Quotient value VR Session 1/VR Session 5	p-value^a^	Quotient value VR Session 5/Eyes Open
**Anteroposterior**				
Total	**<0**.**001**	**2**.**65**	**<0**.**001**	**5**.**68**
**<**0.1 Hz	**0**.**001**	**2**.**84**	**<0**.**001**	**3**.**94**
>0.1 Hz	**<0**.**001**	**2**.**52**	**<0**.**001**	**10**.**27**
**Lateral**				
Total	**0**.**001**	**2**.**02**	**<0**.**001**	**11**.**28**
**<**0.1 Hz	**0**.**021**	**1**.**58**	**0**.**001**	**4**.**97**
>0.1 Hz	**<0**.**001**	**2**.**21**	**<0**.**001**	**21**.**07**

^a^The notation “<0.001” means that the p-value is smaller than 0.001.

**Figure 3:**
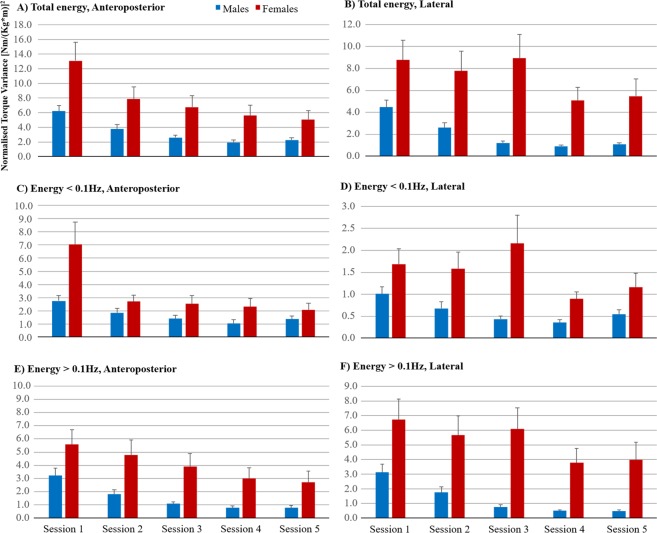
Stability during repeated VR sessions presented individually for males and females. Normalised torque variance values during repeated VR stimuli tests for the male and female groups (mean and SEM values). Within all spectral ranges investigated, both groups gradually reduced the energy used to handle the VR stimuli, but the females on average used particularly more high frequency energy and energy in lateral direction than males.

Post-hoc analysis of general effects of gender revealed no significant difference between genders in any direction or test (Fig. 3).

### Adaptation to repeated VR stimuli

When investigating the adaptation capacity in the entire test subject group (i.e., energy used in VR session 1 vs. VR session 5), the improvements in stability recorded in terms of reduced energy used between the first and last VR sessions were significant in both anteroposterior (p ≤ 0.001) and lateral (p ≤ 0.021) directions (Fig. 2, Table 2). On average in all spectral ranges, the energy reduction was 62% in anteroposterior and 47% in lateral direction. However, when comparing the performance reached after adaptation during VR session 5 with the quiet stance eyes open test, i.e., the control test where the same level of sensory information is available, the energy used remained significantly higher in both anteroposterior (p < 0.001) and lateral (p ≤ 0.001) directions. The energy used was higher in the high frequency range in both anteroposterior and lateral directions during VR session 5 by factors of 10.3 and 21.1 respectively.

As part of the post-hoc analysis of the adaptation processes, exponential regression analyses were performed to evaluate the properties of the adaptive changes to the VR stimuli in males and females (Table 3). The regression analysis revealed a significant adaptation in the male group in total (p < 0.001), low frequency (p = 0.006) and high frequency (p < 0.001) energy used in anteroposterior direction, and in total (p < 0.001), low frequency (p = 0.007) and high frequency (p < 0.001) energy used in lateral direction. The female group only presented a significant adaptation in the anteroposterior direction in the total (p = 0.020) and low frequency (p = 0.008) energy used. In the cases where the adaptation process was significant among females, the time constant was about the same as found among males but the constant values in the regression models were much larger in females than males. Hence, when present, the rate of adaptation was about the same in males and females but the initial performance was worse in females.

**Table 3:** Regression analysis of the effects of Repetition and Gender on the stability.

A. Exponential regression analysis of Torque variance	Gender	p-value^a^	Constant	Time constant
			Energy^b^	Energy/repetition^c^
**Anteroposterior**				
Total	M	**<0.001 [17.7]**	**5**.**89**	**−0**.**28**
	F	**0.020 [5.8]**	**9**.**31**	**−0**.**24**
<0.1 Hz	M	**0.006 [8.2]**	**2**.**42**	**−0**.**23**
	F	**0.008 [7.8]**	**4**.**26**	**−0**.**27**
>0.1 Hz	M	**<0.001 [27.6]**	**3**.**07**	**−0**.**35**
	F	0.051 [4.0]	4.10	−0.24
**Lateral**				
Total	M	**<0**.**001 [28**.**0**]	**4**.**12**	**−0**.**36**
	F	0.205 [1.7]	5.53	−0.18
<0.1 Hz	M	**0**.**007 [8**.**0**]	**0**.**83**	**−0**.**22**
	F	0.206 [1.6]	1.25	−0.15
>0.1 Hz	M	**<0**.**001 [34**.**7**]	**3**.**05**	**−0**.**46**
	F	0.170 [1.9]	3.88	−0.22

^a^p-values and [F-values]. The notation “<0.001” means that the p-value is smaller than 0.001.

^b^Normalised torque variance values, regarding anthropometrical differences between subjects. The parameter reflects the energy used towards the support surface during the first VR session performed.

^c^The exponential regression parameter reflects the average rate by which the energy used towards the support surface is reduced each time the VR session was repeated.

### Presence of near fall events during VR sessions

**Figure 4:**
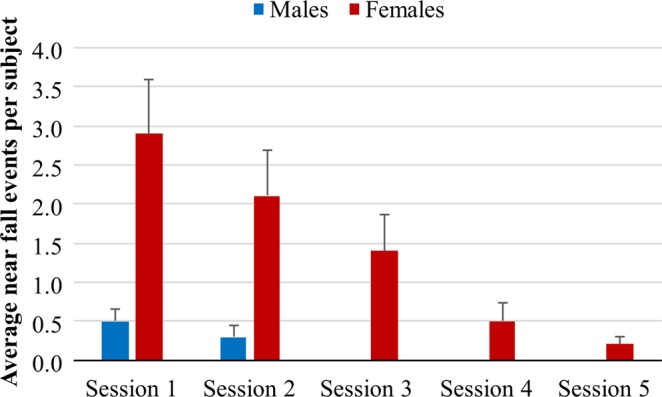
Average near fall events per subject during each VR session in males and females. A near fall event is defined as an event where the study supervisors regarded it necessary to provide tactile support to prevent a fall (mean and SEM values). The near fall events were more common in females but the data also reveals an adaptation process in which instability was reduced with each repetition of the VR stimuli.

A linear regression analysis of the near fall events per subject revealed that repeating the VR sessions significantly reduced the fall events both in males (p = 0.004, [F-value 9.0]) and in females (p = 0.001, [F-value 11.8]) (Fig. 4, Table 4). However, as reflected by the regression constant values, the female subjects experienced initially more near fall events per subject (on average = 3.52) than males (on average = 0.55). The movement direction of the near falls was primarily lateral or slightly diagonal with a lateral direction dominance.

**Table 4:** Regression analysis of the effects of Repetition and Gender on the near fall events per subject.

Linear regression analysis of Near fall events	Gender	p-value^a^	Constant	Time constant
			Near falls^b^	Near falls/repetition^c^
	M	**0**.**004 [9**.**0]**	**0**.**55**	**−0**.**13**
	F	**0**.**001 [11**.**8]**	**3**.**52**	**−0**.**70**

^a^p-values and [F-values]. The notation “<0.001” means that the p-value is smaller than 0.001.

^b^The parameter reflects the average near fall events per subject during the first VR session performed

^c^The linear regression parameter reflects the by which rate the average near fall events per subject is reduced each time the VR session was repeated.

### Effects of Vision and Gender on the stability during the quiet stance control tests

Vision significantly enhanced the stability during quiet stance in the anteroposterior direction as reflected by lower use of total (p = 0.043) and high frequency energy (p < 0.001)(Table 5). Moreover, vision significantly enhanced the stability during quiet stance in the lateral direction as reflected by lower use of total (p = 0.008) and high frequency energy (p = 0.004).

**Table 5:** General effects on the stability during Quiet stance.

Quiet stance stability^a^	Vision^b^	Gender^b^	Vision x Gender^b^
**Anteroposterior**			
Total	**0**.**043 [4**.**8**]]	0.780 [0.1]	0.928 [0.0]
**<**0.1 Hz	0.263 [1.3]	0.790 [0.1]	0.863 [0.0]
>0.1 Hz	**<0**.**001 [26**.**7**]	0.741 [0.1]	0.665 [0.2]
**Lateral**			
Total	**0**.**008 [9**.**1**]	0.666 [0.2]	0.642 [0.2]
**<**0.1 Hz	0.065 [3.9]	0.452 [0.6]	0.244 [1.5]
>0.1 Hz	**0**.**004 [10**.**7**]	0.634 [0.2]	0.206 [1.7]

^a^Repeated measures GLM ANOVA analysis of how the quiet stance stability were affected by main factors “Vision” and “Gender” alone and by their main factor interactions.

^b^p-values and [F-values]. The notation “<0.001” means that the p-value is smaller than 0.001.

Post-hoc analysis confirmed better stability with eyes open than with eyes closed by the significant lesser high frequency (p < 0.001) energy used in anteroposterior direction, and by the significant lesser total (p = 0.011), low frequency (p = 0.036) and high frequency (p = 0.004) energy used in lateral direction (Fig. 2).

## Discussion

In an unfamiliar environment, we tend to make judgments and decisions based on previous experiences and sensory cues in order to adapt^10,11^. The nature of this adaptation has not been studied in detail across repeated sessions of virtual reality. In this study, we demonstrated that watching a VR movie that induced provocative visual motion caused strong balance perturbations during the first session resulting in an energy usage towards the support surface by about 26 (anteroposterior direction) to 47 (lateral) times larger high frequency energy than the levels recorded during the quiet stance control test with eyes open. The quantitative effects of the VR stimuli used on the stability in anteroposterior and lateral directions is described in Table 2. However, repeatedly watching the same VR movie induced a fast multidimensional adaptation of postural control that had reduced the energy used in both directions during the fifth session by 62% (anteroposterior) and 47% (lateral) compared with the first session. This finding suggests that anticipatory and programmed motor activities were activated by visual motion produced by VR by rapidly updating internal models^12,13^. The time constants of the anteroposterior and lateral adaptation processes were about the same (Table 3). This suggests that the CNS can perform an adaptation of the stability processes in parallel in multiple directions, although the biomechanical constraints and the effects of the balance perturbations may have to be addressed differently. A rationale for this approach is that handling the stability of a complex biomechanical structure that is multi-segmented in several directions, means that the appropriate motor actions and adaptations to be made cannot be determined properly from using only an unidirectional stability context.

That said, we found a larger destabilizing effect in lateral direction from VR and this instability was handled more poorly in terms of slower adaptation, especially by females. However, both males and females watched the same VR movie in all five VR sessions. In the quantifying evaluation of the VR stimuli, the VR movie used in the study caused more strain to the stability in the lateral direction compared with the anteroposterior direction (47 times vs 26 times high frequency energy increase at session 1 compared with the quiet stance control test with eyes open). Moreover, the movement direction of the near falls was primarily lateral or slightly diagonal with a lateral direction dominance. Although an overall decrease in low frequency energy used in lateral direction was found between the first and fifth VR sessions, there were fluctuations in the lateral adaptive response and a weaker time constant (Table 3), particularly in females. Hence, the findings raise questions about whether visual motion in a lateral direction in general is more destabilizing or whether this relates to the VR movie used in this study. However, the VR stimuli used cannot explain alone why males and females responded so differently to the same VR stimuli.

The demonstration of reduced use of high frequency anteroposterior and lateral energy with repeated VR sessions is concordant with clinical studies, which have employed VR to enhance motor learning in dysfunction^14^, however not always utilizing motor learning principles^15^. High frequency movement is considered ballistic and is associated with reflexive postural responses^16,17^ and therefore the contribution of high frequency energy is a specific predictor of fall risk to perturbations^18,19^. The reduction of high frequency energy used after repeated VR sessions thus demonstrates a reduced fall risk. Emphasising this point, in a controlled clinical trial Mirelman and colleagues^14^ found that retention effects were more common in treadmill training with VR in patients with Parkinson’s disease, especially for motor and motor-cognitive functions (e.g., gait, obstacle negation and physical performance).

VR technology is becoming popular within the gaming industry. Our study showed that the visual motion in the VR stimuli initially produced marked postural instability, but repeated sessions with VR reduce this instability. The increased postural instability is likely to be the result of an ambiguity between visual information and body movement. In certain contexts, visual motions might be perceived as the most reliable sensory cue, and thus, visual motions regarded as self-motion^20^. A factor that probably promoted a fast adaptation and sensory reweighting in our study is that the subjects were standing on a solid surface in the VR experiments. Hence, the proprioceptive and mechanoreceptive systems provided a stationary motion cue, presumably leading to a switching of self-motion sensation to visual-motion, i.e., VR illusions went from one being a passenger on a rollercoaster ride to one watching a movie of a rollercoaster ride. Thus, prolonging a gaming experience using VR may require disrupting or reducing the information from the other postural control sensory systems by sitting in a chair. Another option could be to make synchronized movements of the chair linked to the activity displayed in the VR movie, thus producing coherent motion signals from both vision and the proprioceptive/mechanoreceptive receptor systems.

Another main finding was that the rate of adaptation to VR was slower in females compared to males when the VR sessions were repeated. Moreover, half of the females handled the VR stimulation quite poorly throughout all repeated sessions compared with males (Fig. 3). However, this poor performance was not consistent in all females, as confirmed by the statistical analyses, because half of the females handled the VR stimulation similarly to males. Moreover, during quiet stance there were no differences in stability related to gender when standing with eyes open and eyes closed. Hence, the gender differences found were related to VR. Similarly, Raper *et al*. have also shown that females have a poorer ability to utilize other spatial information sources when vision is compromised^21^. Although motion sickness was not specifically evaluated in the current study, which could be viewed as a limitation, our results are concordant with those produced by Munafo and colleagues^9^. In their VR experiment within a motion-inducing setting, motion sickness was reported by 56% of participants, and the incidence in women (77.78%) was significantly greater than in men (33.33%)^9^. The authors also reported that individuals who expressed high levels of low frequency lateral postural sway were the most likely to experience motion sickness in VR. This finding suggests that motion sickness is associated with the spectral composition of postural control, where the postural and visual perceptive systems may oscillate at similar frequencies^7,8^. The idea that postural stability and motion sickness are connected in provocative environments is concordant with the views of Riccio and Stoffregen that humans “become sick in situations in which they do not possess (or have not yet learned) strategies that are effective for the maintenance of postural stability”^20^. This is echoed by recent research that suggests that open-loop presentations of motion (particularly self-motion) can be nauseogenic in that they do not allow for dynamic adaptation^22^.

When participants were repeatedly disturbed by the visual perturbation^23^, the energy used towards the support surface gradually decreased through adaptation. However, a striking observation was the use of high frequency anteroposterior and lateral energy under VR, which adapted with session repetition. This high frequency energy adaptation to the conflicting sensory environment in VR is likely associated with a need to suppress the visual motion inputs to postural control^24^, i.e., to use the strategy for standing with eyes closed. Hence, as suggested by Akizuki H *et al*.^4^, in a VR environment where we are faced with incongruent visual motion to actual motion cues, non-vision based receptor systems will eventually prove more reliable, such as the mechanoreceptive and proprioceptive receptor systems which both provide high frequency stability information. The role of the vestibular system with VR is more unclear. The dominant high frequency activity recorded by the force platform is too fast and too poorly associated with head movements to support motor control based on vestibular inputs, partly because the movements of the upper body are increasingly more affected by the movement of inertia^25^.

Interestingly, the low frequency lateral energy used in VR showed lower postural adaptation between sessions, which is in line with the theory that provocative visual motion produces low frequency postural oscillations^26^. Imposed accelerations above 0.2 Hz are usually perceived as one translating through space, whereas accelerations below 0.2 Hz are usually perceived as a change in the direction of the gravito-inertial acceleration vector i.e., a tilt with respect to the gravitational vertical, which may explain the directional effect in terms of lateral sway^6^. However, our findings are not consistent enough to support the hypothesis that subjects less resilient to VR display more low frequency postural movements when standing upright^6^. Moreover, the stability issues and gender differences were primarily reflected by a higher level and poorer adaption of the high frequency postural sway.

A final, and noteworthy finding, was the increased torque variance in post-VR stance (i.e., increased use of energy also after the end of the VR stimuli, see Fig. 1). This postural instability could be the result of a change in strategy during VR where the robustness of postural control is enhanced by switching from the more energy efficient vision-based sensory cues to the less energy efficient but more reliable proprioceptive/mechanoreceptive sensory cues for postural control. An alternative possibility might be that it is an aftereffect reflecting the continued expression of a learnt behaviour to handle VR.

Clinically, a large proportion of patients with chronic dizziness experience visual vertigo^27^, a symptom characterised by poor resilience to visual motion. Follow-up studies in this population have suggested that high visual sensitivity significantly impedes quality of life^28^ but visual desensitisation training, through repeated sessions with optokinetic stimuli, suppresses symptoms^29^ through cortical re-modelling^30^. However, these studies have not involved a detailed investigation of postural changes. Here, we provide evidence to suggest that VR could be a potential therapeutic stimulus for patients with visual vertigo, as repeated sessions with VR reduce postural sway in visually provocative environments. The idea of using VR in balance therapy has the potential to revolutionise rehabilitation in patients with chronic dizziness; a population that is currently very difficult to treat and manage. Smaller responses during VR presumably correspond to reduced visual motion sensitivity, an outcome that will add confidence to patients with visual vertigo. Furthermore, VR may be a preferred option for some patients over optokinetic training, because it can be performed at home, is engaging and interactive, and more closely mimics real-life situations^31,32^. However, females may not be able to adapt as effectively in a lateral direction and may benefit from other modes of balance rehabilitation.

A limitation of this study is that the investigated population included no patients with visually induced balance disorders. The scope of this study was to investigate how normal subjects handle VR stimuli and whether the CNS could sustain a simultaneous, multidimensional adaptation while submitted to multidimensional visual motion. Intriguingly, while the response pattern tended to be uniform among normal healthy males, it was not in females. Thus, if VR is used in clinical settings certain flexibly may have to be applied in terms of intensity and duration of the treatment programs. For example, one may have to design specific VR movies to address certain disorders.

To conclude, healthy human subjects can handle a multidimensional provocative visual environment after 4–5 repeated practices with VR, and initiate an adaptation that markedly reduces the energy used for stability control in both anteroposterior and lateral directions. Consequently, VR technology might be an effective tool for rehabilitation. However, some females may require more training sessions to achieve effects with VR.

## Methods

Experiments were performed in accordance with the Helsinki declaration and approved by the Scientific Ethical Committee at Lund University, Sweden (Dnr 2018-320). All participants provided written informed consent before the testing commenced.

### Subjects

Twenty healthy, consenting, subjects participated in the study (10 females; mean age 27.2 years (SD 8.8); mean height 1.76 meters (SD 0.10); mean mass 73.8 kilograms (SD 14.0)). The subjects were recruited from the student and staff population and they received no fee for participating. Exclusion criteria were lesions or disorders of the sensorimotor system, previous postural or vertiginous conditions such as vestibular migraine, or major lower limb fracture in the previous year. All subjects had normal or corrected to normal visual acuity using glasses or contact lenses. Subjects were not under the influence of alcohol or drugs.

To determine the influence of gender on postural control and sensorimotor adaptation during the tests, a subgroup analysis was also performed between males and females. The female subgroup included 10 subjects of mean age 25.9 years (SD 8.8); mean height 1.67 meters (SD 0.05); mean mass 64.6 kilograms (SD 8.8), and the male subgroup included 10 subjects of mean age 28.5 years (SD 9.1); mean height 1.84 meters (SD 0.06); mean mass 82.9 kilograms (SD 12.2).

### Posturography assessment

A custom-built force platform recorded torques and shear forces with six degrees of freedom using force transducers with an accuracy of 0.5 N. A customized program sampled the force platform data at 50 Hz. The virtual reality stimulation was produced by a HTC Vive™ system presenting the VR movie Desert Ride Coaster by Speed Scream™. The VR movie was 90 seconds long and is available for download on the HTC Vive website. In brief, it contained a simulation of travelling on a railroad track situated in a mountainous terrain, moving forward uphill, downhill or moving on flat surface while the railroad track made irregular turnings to the left and right. Hence, the provocative visual motion was marked in both anteroposterior and lateral directions. Each participant stood without shoes on the force platform in an upright, but relaxed posture with arms alongside the body, heels 3 cm apart and feet positioned at 30° along guidelines on the platform.

All subjects performed seven tests in a fixed order:

(1). Quiet stance with eyes open (EO).

(2). Quiet stance with eyes closed (EC).

(3–7). VR stimuli session 1- session 5.

Between all tests, subjects were allowed to remove the VR helmet, if equipped with one, and sit and rest for 3 minutes. During the two initial control tests (1–2), the participants were instructed to focus on a target 1.5 m in front of them at eye level or keep their eyes closed. In order to study the effects of adaptation to VR stimuli, the same VR movie was displayed 5 times, noted as tests (3–7) above. During the VR sessions, subjects listened to the sound accompanied with the VR movie through headphones. All participants were naive to the VR stimulus and were not informed about the effect VR stimuli would have on their balance. The information given to the subjects prior to the VR series was restricted to stating that the VR device would display a movie to them. The information given included no instructions about how to handle any potential instability caused by watching the moving.

Stability was recorded for 120 seconds during all seven tests. During the VR session, the recordings were started about 3 seconds before the 90-second VR movie was started and the recordings continued for about 27 seconds after the VR movie has ended, the VR equipment during this period displaying the last image in the movie as static. Several subjects experienced near fall events mostly during the first two VR sessions, where the study supervisors gave the subjects gentle tactile support before foot lift. No subject needed support during the quiet stance control tests. The number of near fall events, defined as an event where the study supervisors regarded it necessary to provide tactile support to prevent a fall, was counted for each subject and VR session. An evaluation of this data is presented in the result section.

### Analysis

Postural stability was investigated in the anteroposterior and lateral directions by analysing the variance of anteroposterior and lateral torque values. The torque variance values correspond to the energy used towards the support surface to preserve stability^25^, i.e., the efficiency of standing^33^. The torque produced at different sites of the body is the means used for controlling stability^33^. For a detailed explanation on torque and its relationship to standing postural control, see Johansson *et al*.^25^.

Force platform recordings were divided spectrally into total torque variance (total energy), torque below 0.1 Hz (<0.1 Hz; low frequency energy); and torque above 0.1 Hz (>0.1 Hz; high frequency energy) using a fifth-order digital Finite duration Impulse Response (FIR) filter, with filter components selected to avoid aliasing. These spectral separations were done to categorise smooth corrective changes of posture (i.e. <0.1 Hz) and fast corrective movements to maintain balance (i.e. >0.1 Hz)^16^. Clinically, increased fast corrective movements (>0.1 Hz) are commonly associated with decreased visual information^16^ and physiological factors such as being over-weight or fatigued^34^, whereas the low frequency movements (<0.1 Hz) are commonly increased by poor surface conditions like standing on foam^17,35^. Torque variance values were normalised to account for anthropometric differences between subjects, using the subjects’ squared height and squared weight, as height and weight influence body sway^25^. The squared nature of the variance algorithm made it necessary to use normalisation with squared parameters to achieve unit agreement.

### Statistical analysis

The torque variance values during the quiet stance control tests and during the five VR stimuli perturbation sessions were analysed using repeated measures GLM ANOVA on log-transformed values. The main factors and factor interactions analysed were: ‘Repetition’ (Session 1…5; d.f. 4); and ‘Gender’ (Male vs. Female; d.f. 1). For the control tests, the main factors and factor interactions analysed were: ‘Vision’ (Eyes closed vs. Eyes open; d.f. 1); and ‘Gender’ (Male vs. Female; d.f. 1). The repeated measures GLM ANOVA analysis method was used after ensuring that all dataset combinations analysed in the study with this statistical method produced model residuals that had normal distribution, thus validating its appropriateness (i.e., whether the GLM ANOVA method was appropriate for correctly analysing the data)^36^.

The Mann-Whitney U (Exact sig. 2-tailed) test was used for between-groups post hoc comparisons. The Wilcoxon matched-pairs signed-rank test (Exact sig. 2-tailed) was used for within-subjects post hoc comparisons, i.e., analysing the adaptive changes between VR Session 1 and VR Session 5^18^. Moreover, as part of the post hoc evaluation, the best fitting dynamic patterns and time constants describing the changes in energy used (reflecting stability) and average number of near fall events per subject during the five repeated VR sessions over time were determined by using regression models after evaluating different regression models for best fit (linear, exponential etc.). An exponential regression model was found best describing changes recorded in energy used over repeated VR sessions whereas a linear regression model was found best describing the changes in average near fall events per subject over repeated VR sessions.

In all analyses, p-values < 0.05 were considered significant also after Bonferroni correction as in the post-hoc analysis, no dataset was included in within-subject or between-groups tests more than once. The Shapiro-Wilk test revealed that some datasets were not normally distributed and that normal distribution could not be obtained by log-transformation. Thus, non-parametric statistical methods able to appropriately handle direct comparisons of individual datasets of non-normal distribution were used in all post-hoc statistical evaluations^36^.
